# Advances in the Pathogenesis of Auto-antibody-Induced Cerebellar Synaptopathies

**DOI:** 10.1007/s12311-021-01359-z

**Published:** 2022-01-22

**Authors:** Hiroshi Mitoma, Mario Manto

**Affiliations:** 1 Department of Medical Education, Tokyo Medical University, Tokyo, Japan; 2 Unité des Ataxies Cérébelleuses, Service de Neurologie, Médiathèque Jean Jacquy, CHU-Charleroi, 6000 Charleroi, Belgium; 3 Service des Neurosciences, University of Mons, 7000 Mons, Belgium

**Keywords:** Immune-mediated cerebellar ataxias, Anti-GAD antibody, Anti-mGluR antibody, Anti-VGCC antibody, Anti-GluR delta antibody, Long-term depression, Synaptopathy

## Abstract

The presence of auto-antibodies that target synaptic machinery proteins was documented recently in immune-mediated cerebellar ataxias. The autoantigens include glutamic acid decarboxylase 65 (GAD65), voltage-gated Ca^2+^ channel (VGCC), metabotropic glutamate receptor type 1 (mGluR1), and glutamate receptor delta (GluRdelta). GAD65 is involved in the synthesis, packaging, and release of GABA, whereas the other three play important roles in the induction of long-term depression (LTD). Thus, the auto-antibodies toward these synaptic molecules likely impair fundamental synaptic machineries involved in unique functions of the cerebellum, potentially leading to the development of cerebellar ataxias (CAs). This concept has been substantiated recently by a series of physiological studies. Anti-GAD65 antibody (Ab) acts on the terminals of inhibitory neurons that suppress GABA release, whereas anti-VGCC, anti-mGluR1, and anti-GluR Abs impair LTD induction. Notably, the mechanisms that link synaptic dysfunction with the manifestations of CAs can be explained by disruption of the “internal models.” The latter can be divided into three levels. First, since chained inhibitory neurons shape the output signals through the mechanism of disinhibition/inhibition, impairments of GABA release and LTD distort the conversion process from the “internal model” to the output signals. Second, these antibodies impair the induction of synaptic plasticity, rebound potentiation, and LTD, on Purkinje cells, resulting in loss of restoration and compensation of the distorted “internal models.” Finally, the cross-talk between glutamate and microglia/astrocytes could involve a positive feedback loop that accelerates excitotoxicity. This mini-review summarizes the pathophysiological mechanisms and aims to establish the basis of “auto-antibody-induced cerebellar synaptopathies.”

## Introduction

Autoimmunity affects the cerebellum, leading to the manifestations of the cerebellar ataxias (CAs), termed immune-mediated cerebellar ataxia (IMCAs). IMCAs encompass diverse etiologies and pathophysiological mechanisms [1–9]. Autoimmunity can be triggered by another pathology in some patients, such as infection (post-infectious cerebellitis: PIC), neoplasm (paraneoplastic cerebellar degeneration: PCD), and gluten sensitivity (GA), although the triggering factor is some patients remains obscure (primary autoimmune cerebellar ataxia: PACA) [2, 6, 7]. Despite their immune diversity, the majority of IMCAs is commonly associated with auto-antibodies against cerebellar autoantigens. Notably, some of these antigens are involved in cerebellar synaptic transmissions, which include glutamic acid decarboxylase 65 (GAD65), voltage-gated Ca2^+^ channel (VGCC), metabotropic glutamate receptor type 1 (mGluR1), and glutamic receptor delta (GluR delta) [10, 11]. Influx of Ca^2+^ through the VGCC is the first step in transmitter vesicle release from the presynaptic terminals [12], and, at the level of GABAergic terminals, GAD65 is involved in GABA synthesis and its packaging into synaptic vesicles [13]. Notably, VGCC, mGluR1, and GluR delta on Purkinje cells (PCs) are involved in the induction of long-term depression (LTD) between parallel fibers (PFs) and PCs, a critical form of synaptic plasticity in the cerebellum [10, 11].

Auto-antibodies towards ion channel- and synapse-related molecules have been also identified in autoimmune limbic encephalitis [14–18]. Auto-antibodies induced neurological diseases show various clinical phenotypes (see details in Table 1). Among these diverse features, it should be acknowledged that auto-antibodies toward glutamate receptors, GABA receptors, and K^+^ channel-related proteins are preferentially found in autoimmune limbic encephalitis but not in IMCAs [10] (Table 1). In autoimmune limbic encephalitis, it is assumed that these auto-antibodies diffusely interfere with basal synaptic transmission or neural excitability and weaken overall functions of the temporal lobe [18].

**Table 1:** Auto-antibodies toward ion channel- and synapse-related antigens commonly found in immune-mediated cerebellar ataxias and autoimmune limbic encephalitis

Auto-antibody	Target epitope	Autoimmune trigger	Frequency and features of immune-mediated cerebellar ataxias	Frequency and features of autoimmune limbic encephalitis	Other phenotypes
Ion channels and related proteins					
Anti-LGI1	-Leucine-rich glioma-inactivated 1 (LGI1), one of the voltage-gated potassium channel (VGKC) Kv1 complex	-Non-paraneoplastic	Rarely associated -20% of the patients with limbic encephalitis developed motor symptoms including CAs.	**Common phenotyp**e -Men in their 60’s -Amnesia, confusion/disorientation, seizures	–
Anti-Caspr2	-Contactin-associated protein-like 2 (Caspr2), an associated protein of VGKC Kv1	-Paraneoplastic (thymoma), 20%, -Non-paraneoplastic	Sometimes associated -35% of the patients with limbic encephalitis or Morvan syndrome developed CAs. -Some patients with limbic encephalitis showed episodic CAs.	**Common phenotyp**e -Men in their 60’s -Limbic encephalitis is the most common phenotype, cognitive disturbance seizures	-Morvan syndrome is characterized by central, peripheral and autonomic hyperexcitability (limbic encephalitis, insomnia peripheral nerve hyperexcitability, neuropathic pain, autonomic dysfunction, weight loss)
Anti-DPPX	-Dipeptidyl-peptidase-like protein-6 (DPPX), an auxiliary subunit of VGKC Kv4.2	-Non-paraneoplastic	Sometimes associated -CAs are one of multifocal neurological symptoms -A case with pure CAs and myoclonus was reported.	**Common phenotype** -Men in their 50’s -Agitation, delusions, hallucinations, myoclonic jerks, seizures	-Association of brainstem disorder (dysphagia, dysarthria, respiratory failure), and autonomic failure
Anti-VGCC	-P/Q-type voltage-gated calcium channel (VGCC)	-Paraneoplastic (SCLC) mostly, -Non-paraneoplastic	**Common phenotyp**e -50–60’s -Pancerebellar ataxias	Not documented	-Association of Lambert-Eaton myasthenic syndrome
Synaptic release machinery proteins					
Anti-GAD65	-Glutamic acid decarboxylase 65	-Idiopathic, -A few in autoimmune conditions such as paraneoplastic and gluten sensitivity	**Common phenotype** -Women, in 60’s -Gait ataxia associated with variable degree of limb ataxia and dysarthria	**Sometimes associated** - Cognitive disturbance, seizure	-Oculomotor deficits -Stiff-person syndrome The overlap syndromes are observed during long follow-up.
Synaptic adhesion/organizing molecules					
Anti-GluR delta	-Glutamate receptor delta	-Infection -Vaccination	**Common phenotype** -Children -Gait ataxia associated with variable degree of limb ataxia and dysarthria	Not documented	-
Receptors					
Anti-AMPA-R	-GluR1,2,3 unit	-Paraneoplastic (SCLC, Breast cancer, thymoma) -Non-paraneoplastic	Rarely associated 14% of the patients developed CAs.	**Common phenotype** -Middle to aged women -Behavioral change, memory loss	
Anti-NMDA-R	-NR1-NR2 unit	-Paraneoplastic (ovarian teratoma) -Non-paraneoplastic	Very rare Atypical symptoms such as cerebellar ataxia or hemiparesis predominated in children.	**Common phenotyp**e -Young women -Psychosis, seizures	–
Anti-mGluR1	-Metabotropic glutamate receptor type 1	-Paraneoplastic (Hodgkin’s lymphoma, cutaneous T lymphoma, prostate adenocarcinoma) -Non-paraneoplastic	**Common phenotype** -In 40–60’s -Gait and limb ataxia	Not documented	-Association of behavioral changes (irritability, apathy, mood, personality change, psychosis with hallucinations, and catatonia), cognitive changes (memory deficit, deficits in executive functions and spatial orientation) or dysgeusia
Anti-GABA_A_-R	-GABA_A_ R, α1, and β3 subunits	-Paraneoplastic (thymoma), -Non-paraneoplastic	Very rare	**Common phenotype** -Children, adults -Seizures, memory and cognitive deficits, behavioral changes, psychosis	–
Anti-GABA_B_-R	-GABA_B_ R, B1 subunit	-Paraneoplastic (SCLC) -Non-paraneoplastic	Very rare	**Common phenotype** -In 30–70’s -Seizures, confusion memory loss	–
Anti-glycine-R	-Glycine R	-Paraneoplastic -Non-paraneoplastic	Rarely associated 13% of the patients developed CAs.	Not documented	-Progressive encephalomyelitis with rigidity and myoclonus (PERM) is characterized by stiffness of the axial and lower limb muscles, brainstem signs, and hyperlplexia (brainstem myoclonus or excessive startle)

*CAs* cerebellar ataxias, *R* receptor, *SCLC* small cell lung cancer. Modified from Mitoma et al. (2020) [10]

Each region in the central nervous system is endowed with particular synaptic machinery types of proper properties for delivering the region-specific functions. Impairments in these crucial synapses lead to the loss of the region-specific functions. Thus, it is likely that in addition to diffuse deterioration in basal synaptic transmission, auto-antibodies in the cerebellum potentially impair the local particular synaptic machinery, resulting in the loss of specific cerebellar functions.

This mini-review aims to clarify how auto-antibodies impair the cerebellar particular synaptic machineries, so as to induce loss of cerebellar specific functions. For this goal, we provide an overview of the current knowledge of the clinical profiles of CAs associated with anti-GAD, anti-VGCC, anti-mGluR1, and anti-GluR delta antibodies (Abs) and the physiological actions of these Abs (see “Anti-GAD Antibody” and “Anti- VGCC, mGluR1, and GluR Delta Antibodies” sections). Based on this background, we then discuss the pathophysiological mechanisms underlying various auto-antibodies-induced CAs (see the “Relevance of Synaptic Dysfunction with Impairments in Internal Forward Model” and “Structural Vulnerability of the Cerebellum and Neuroinflammation” sections): (*1*) these auto-antibodies disorganize the machinery of GABA-mediated disinhibition/inhibition so as to distort the conversion process from the internal model, a model that emulates the dynamics of body and environments internally in the brain, to cerebellar output signals, (*2*) they also disorganize the induction of synaptic plasticity, resulting in loss of restoration and compensation of the internal model and subsequent amplification of CAs, and (*3*) these functional synaptic disorders can induce excitotoxicity, leading to neuroinflammation that serves as “the pathological transmitter-immunity cycle.” In the “Conclusion” section, we discuss the future experiments which could further help in the unravelling of the synaptic/system consequences of immune attacks triggered by auto-antibodies.

## Anti-GAD Antibody

GAD is an enzyme that catalyzes the conversion of glutamate to GABA [19, 20]. It exists in two isoforms: GAD65 and GAD67. In 2001, Honnorat and colleagues reported a first series of 14 cerebellar patients associated with high titer of anti-GAD65 Ab [21]. This is the first report highlighting the importance of antibodies targeting a key-enzyme in GABA synthesis.

### Clinical Profiles of Anti-GAD Ataxia

Anti-GAD ataxia is defined as sporadic cerebellar ataxia associated with high titers of anti-GAD65 Ab in both the serum and cerebrospinal fluid (CSF) [19–23]. Anti-GAD65 Ab is produced intrathecally. Levels of serum anti-GAD65 Ab titers are usually more than 2000 U/mL, or 10- to 100-fold those in patients with type 1 diabetes mellitus (T1DM) [19–23]. The triggering factor of autoimmunity is usually not apparent. However, in some cases, autoimmunity is triggered by neoplasm and gluten sensitivity [20]. Anti-GAD ataxia is often associated with other autoimmune diseases, such as T1DM, autoimmune thyroid diseases, and pernicious anemia [19–23].

Although Ab- or cell-mediated autoimmunity towards GAD65 does not affect the cerebellum only but the entire CNS, the cerebellum is one of the most vulnerable areas [19–23]. Thus, anti-GAD ataxia is sometimes associated with extracerebellar symptoms, including temporal lobe epilepsy, limbic encephalitis, ophthalmoplegia, opsoclonus, and stiff-person syndrome (SPS) [19–23]. The overlap syndromes are observed during long follow-up in 14-36% of the CA patients [23].

Anti-GAD ataxia affects mostly women in their 60s and exhibits either subacute or chronic/insidious onset [19–22] (Table 2). It is still unclear whether patients show a prodromal phase. Patients often present with gait ataxia and a variable degree of limb ataxia and scanning speech [19–22]. MRI shows normal aspect of the cerebellum or vermian involvement depending on the duration of illness [19–22], suggesting that cell degeneration occurs depending on the disease progression. CSF studies sometimes show oligoclonal bands [19–22].

**Table 2:** Clinical profiles of anti-GAD65, anti-VGCC, anti-mGluR and GluR delta Abs-associated cerebellar ataxias

	Anti-GAD	Anti-VGCC	Anti-mGluR1	Anti-GluR delta
Prevalence in IMCAs*	2%	Prevalence of PCD in IMCAs: 3% Frequency among PCD: 2%	Rare	Rare
Trigger of autoimmunity	Unknown	Mainly with paraneoplasia (SCLS, prostate adenocarcinoma, non-Hodgkin’s lymphoma). A few without paraneoplasia	Some with paraneoplasia (Hodgkin’s lymphoma, prostate adenocarcinoma). Others without paraneoplasia or infection	Infection, vaccination
Age, gender	60s, females (mostly)	50–60s	Median 55 years (IQR 43–64), 43% females	Children
	Insidious and chronic or subacute	Subacute	Subacute	Acute
Features of CAs	Gait ataxia associated with variable degree of limb ataxia and dysarthria. Sometimes associated with stiff-person syndrome or epilepsy.	Pancerebellar ataxias with or without Lambert-Eaton syndrome	Gait and limb ataxias. Sometimes associated with extracerebellar symptoms (behavioral and cognitive changes)	Gait ataxia associated with variable degree of limb ataxia and dysarthria
MRI	Present depending on duration of ataxia	Not at onset but may develop rapidly	Variable: From no to mild atrophy	No atrophy
Therapeutic outcomes	Variable. From good response to poor response to corticosteroids, IVIg, immunosuppressants, PE, and rituximab.	Paraneoplasia: Variable. From good to poor response to IVIg, corticosteroids, and mycophenolate mofetil. Non-paraneoplasia: Improvement.	Paraneoplasia: Variable. From good response to poor response to IVIg, PE. Non-paraneoplasia: Generally good response to IVIg, corticosteroids, mycophenolate, cyclophosphamide, and rituximab.	Generally good response to IVIg or IVMP.

*Abs* antibodies, *IMCAs* immune-mediated cerebellar ataxias, *PCD* paraneoplastic cerebellar degeneration, *SCLS* small cell lung cancer, *IVIg* intravenous immunoglobulins, *IVMP* intravenous methylprednisolone, *PE* plasma exchange. The prevalence (asterisk) is cited a reference Mitoma et al. (2016) [2]

Immunotherapies encompass two steps depending on the purpose: induction therapy and maintenance therapy. Induction therapy is used to minimize CAs at short-term in a rapid fashion [19, 22]. Various immunotherapeutic agents, either alone or in combination, are recommended until remission. Maintenance therapy is used to prevent relapse [19, 22]. Both types of therapies include corticosteroids, intravenous immunoglobulins (IVIg), immunosuppressants, plasmapheresis, and rituximab, either alone or in combination [19, 22]. Up until now, there are no reports of any significant differences in the therapeutic benefits from these types [19, 22].

### Synaptic Actions of Anti-GAD Ab

**Fig. 1:**
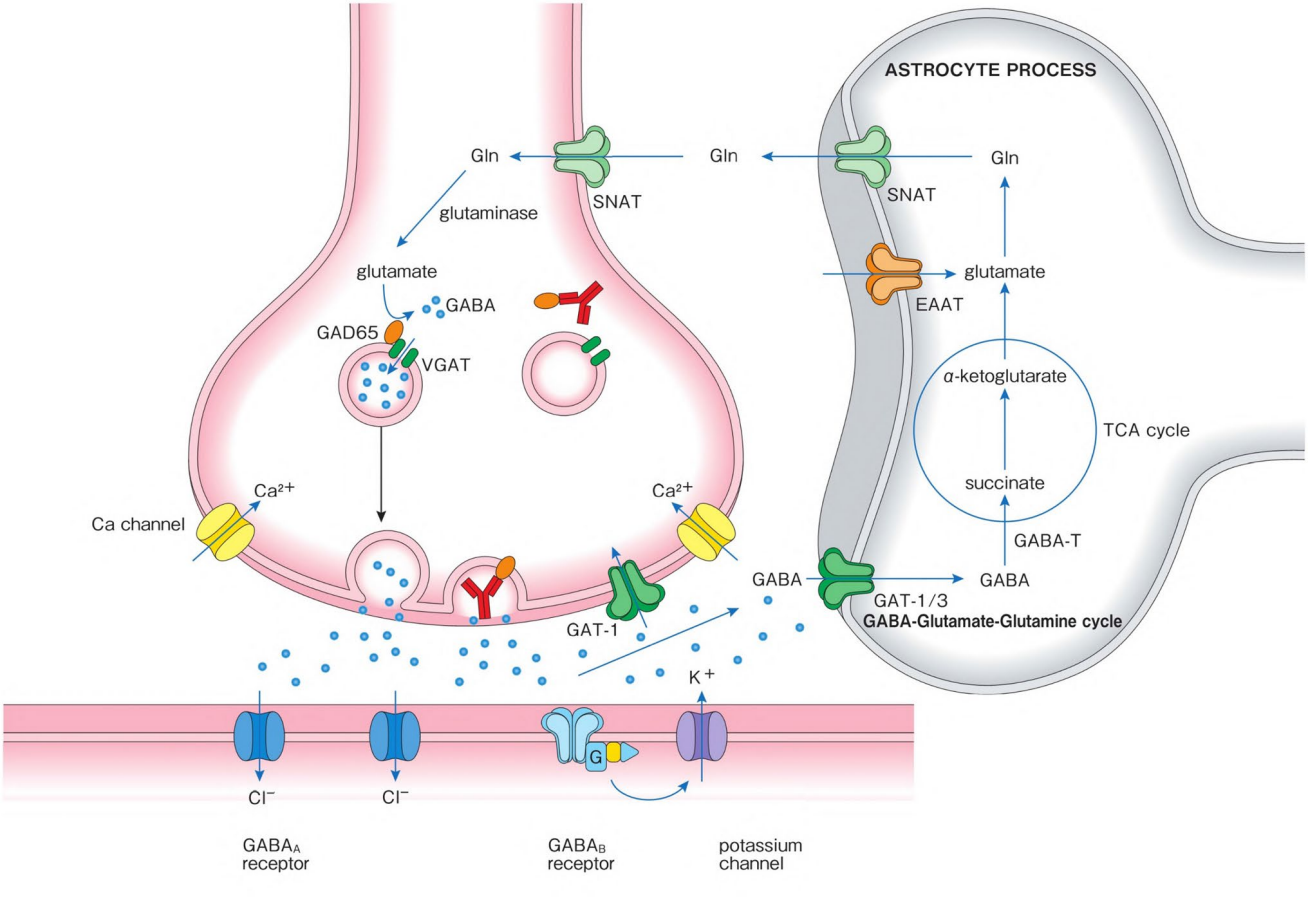
Pathogenic actions of anti-GAD65 antibody. Anti-GAD65 antibody (Ab) can be internalized, presumably during exocytosis or endocytosis. Since GAD65 is assumed to be exposed during exocytosis, anti-GAD65 Ab have access to this antigen. Anti-GAD65 Ab disturbs the association of GAD65 with the synaptic vesicles, which results in impairment of GABA packaging into the vesicles and shuttling of vesicles to their release sites. The decrease in GABA release impairs cerebellar signal formation, leading to disorganized cerebellar controls. Importantly, such pathogenic action by anti-GAD65 Ab is epitope dependent

The significance of anti-GAD65 has been a matter of debate [19, 20, 24]. Some researchers have argued that anti-GAD65 Ab has no pathogenic roles in the development of CAs based on the following reasons [25–27]: (*1*) Anti-GAD65 Ab is nonspecific and found in T1DM and various neurological conditions, such as SPS. (*2*) If the cause is solely due to anti-GAD65 Ab, the anti-GAD65 Ab increases in titer as the disease progresses. However, there is no correlation between anti-GAD65 Ab titer and clinical features. (*3*) GAD65 is intracellularly located, implying that anti-GAD65 Ab does not have a direct access to GAD65. (*4*) Most importantly, the application of CSF IgGs using cerebroventricular, intrathecal, and intraperitoneal (with blood-brain barrier permeabilization) methods in in vivo preparations did not impair cerebellar functions. Thus, there has been no evidence of passive transfer experiments. In contrast, recent rodent experiments have demonstrated impairment of cerebellar-mediated modulations following intracerebellar application of CSF IgGs, which was confirmed by various types of experiments, including excitability of the spinal cord or motor cortex, gait, behavioral tasks, and blink reflexes [28–30]. These passive transfer experiments are highly suggestive for the pathogenic effects of anti-GAD65 Ab in CAs. Furthermore, the synaptic and molecular mechanisms underlying these anti-GAD65 Ab-induced pathogenic actions have been clarified (Fig. 1). Nevertheless, it should be acknowledged that these studies did not rule out secondary involvement of cell-mediated mechanisms.

#### Decrease in GABA Release Following the Binding of GAD65 with Anti-GAD65 Ab

Studies using slice-tissue preparations showed that the addition of CSF IgGs from patients with anti-GAD ataxia to the perfusion medium elicited presynaptic inhibition of GABAergic synapses between basket cells and PCs so as to decrease GABA release [31, 32]. Importantly, these pathogenic actions of IgGs from patients with anti-GAD ataxia were elicited by the binding of GAD65 with anti-GAD65 Ab itself; these actions were abolished after absorption of anti-GAD65 Ab with recombinant GAD65 [33] while anti-GAD65 Ab elicited no actions in slices from GAD65 knockout mice where inhibitory transmission was mediated by a compensatory effect of GAD67 [30].

#### Epitope-Specific Actions

Studies using monoclonal antibodies showed that these in vivo and in vitro actions were epitope dependent [29, 30]. Human monoclonal GAD65Ab b78 recognizes an epitope that is recognized by GAD65Ab in patients with CA, whereas human monoclonal GAD65Ab b96.11 binds to a common epitope that is shared by GAD65Ab in patients with T1DM [19]. The b78 monoclonal Ab with epitope specificity in CAs exhibited pathogenic actions, whereas another b96.11 monoclonal Ab in T1DM had no such actions [29, 30]. Consistently, pathogenic actions were found in CSF IgGs from patients with anti-GAD ataxia, not CSF IgGs in T1DM [28, 31]. Furthermore, the actions of CSF IgGs were different even between samples from cerebellar patients and samples from SPS patients; impairment of exocytosis in the former and a decrease in GABA synthesis in the latter [29]. One epitope mapping study that employed competition assay using human monoclonal Ab showed consistent data with physiological data. Patients with SPS recognized the b78-defined epitope significantly better than patients with CA [29], showing that the recognition of b78-defined epitope was different among anti-GAD65 Abs in CA and SPS. Since the identification of disease-specific anti-GAD65 Abs is complicated by the conformational nature of many of these epitopes, epitope mapping should be analyzed by competition assay using human monoclonal Ab, not peptides and deletion mutants [19]. Notably, low titer anti-GAD Ab had no pathogenic actions, suggesting that the low titer anti-GAD65 Ab defined epitope is different from the high titer anti-GAD65 defined epitope [19].

In the cerebro-cerebellar loop, chained GABAergic neurons (Basket cells and PCs) determine the phasic command on timing and synergy (see the “GABA-Mediated Disinhibition/Inhibition Mode for Online Predictive Controls*”* section) for coordination [34], with specific emphasis on the exact timing of GABA release. In contrast, in the spinocerebellar loop [19], GABAergic outputs from PCs might modulate excitatory signals, with specific emphasis on tonic GABA supply. These physiological data suggest that GAD65Ab can elicit either CAs or SPS depending on epitope specificity. However, further experimental studies are required to clarify the short-term, middle-term and long-term consequences on the synaptic machinery.

#### Internalization and Dissociation of GAD65 with Vesicles

Anti-GAD65 Ab is internalized, presumably by exocytosis or endocytosis [30, 35]. Anti-GAD65 Ab impairs the association of GAD65 with vesicles, resulting in deficits in GABA packaging into vesicles and shuttling of vesicles to their release sites [30] (Fig. 1). Thus, physiological studies suggest that anti-GAD65 can access to GAD65. A possible mechanism underlying the access route is that GAD65, attached on the cytosolic face of vesicles, is exposed to the space in vesicles during exocytosis, thereby which anti-GAD65 Ab within vesicles might bind to exposed portion of GAD65 [19, 30]. However, the underlying mechanisms behind this transient and repetitive exposure are still not clear.

#### From Functional Impairment to Cell Death

A decrease in GABA release attenuates the spill-over GABA-induced presynaptic inhibition on glutamate release from neighboring PFs, which elicits major imbalance between GABA and glutamate leading to excitotoxicity [36]. Consistently, one autopsy study revealed the complete loss of PCs [37]. Previous studies also showed that some patients showing no evident cerebellar atrophy respond well to immunotherapies and that the clinical improvement correlates well with a fall in Ab titers [3, 6]. Taken together, these findings indicate that Abs titers better reflect functional disorders rather than cell death. Auto-antibodies-induced functional excitotoxicity induces activation of microglia, resulting in interference of glutamate uptake by astrocytes. Such an amplification might facilitate cell death in the advanced stage (see detailed discussions in the “Structural Vulnerability of the Cerebellum and Neuroinflammation” section).

## Anti-VGCC, mGluR1, and GluR Delta Antibodies

### Anti-VGCC Ab-Associated Cerebellar Ataxia: Clinical Profiles and Actions of Anti-VGCC Ab

The action potential at the presynaptic terminal activates the VGCCs, such as P/Q-type and/or N-type Ca^2+^-channel. Anti-VGCC Abs were first described in 1992 in association with Lambert-Eaton myasthenia syndrome (LESM) [38]. However, the association of auto-antibodies toward the P/Q-type VGCC with CAs was also described in patients with paraneoplastic cerebellar degeneration (PCD) with or without LEMS [38], especially in LEMS-positive patients [39] (Table 2). The reported prevalence of the association of anti-VGCC Ab among PCD patients is 2% [40]. The neurological manifestations in the affected patients are similar to those with PCD: acute or subacute pancerebellar ataxia, sometimes preceded by nausea, vomiting, and dizziness [39]. CSF studies show inflammatory changes, including lymphocytic pleocytosis, increased protein concentrations, high IgG index, and oligoclonal bands [39]. On the other hand, anti-VGCC Ab is also detected in non-paraneoplastic conditions [38]. For example, a large-scale study showed that anti-VGCC Abs were positive in 8 of 67 patients who showed non-paraneoplastic chronic cerebellar degeneration [41].

The therapeutic response depends on the clinical background. The paraneoplastic patients show a poor response to immunotherapies. One possible explanation is that paraneoplastic conditions are associated with persistent exposure to the antigens. The cytotoxic T cell-mediated killing of tumor cells releases additional cancer antigens, resulting in the continuity of the cancer-immunity cycle. One study of 16 anti-VGCC Ab-positive patients with PCD and SCLC [42] reported complete recovery in 1 case, stabilization at a low Rankin score in 5 cases, and stabilization or worsening at high Rankin scores in 5 cases [42]. The median survival time of these patients was 12 months. In contrast, good prognosis was reported in patients with non-paraneoplastic conditions [41]. These immunotherapies included IVIg, corticosteroids, and mycophenolate mofetil [42].

Intrathecal administration of serum IgGs obtained from anti-P/Q-type VGCC-positive patients with PCD and LEMS elicited ataxic symptoms in mice [43]. A polyclonal peptide Ab against the major immunogenic region in P/Q-type VGCC (the extracellular domain-III S5-6 loop) impaired the P/Q-type VGCC and caused a decrease in Ca^2+^ currents, impairing synaptic transmission between PF and PC [44]. However, the actions of anti-VGCC Ab on PF-PC LTD have not been studied so far and deserve specific experiments.

### Anti-mGluR1 Ab-Associated Cerebellar Ataxia: Clinical Profiles and Actions of Anti-mGluR Ab

mGluR 1 is coupled to the G-protein Gq family, which mediates inositol trisphosphate (IP_3_)-induced Ca^2+^ mobilization and activation of protein kinase C (PKC) [45]. The association of CAs with anti-mGluR1 Ab has been reported in both paraneoplastic and non-paraneoplastic conditions [46–50] (Table 2). The main neurological manifestations are subacute gait and limb ataxia, which are sometimes associated with extracerebellar symptoms [48] (Table 2). Anti-mGluR Ab is identified in both serum and CSF. CSF sometimes shows pleocytosis. At the onset, MRI studies usually show no atrophy, but sometimes detect T2/FLAIR hyperintensities or leptomeningeal gadolinium enhancement [48]. However, the MRI shows cerebellar atrophy as the disease progresses, confirming a neuronal loss and deleterious effects on the cerebellar reserve. The beneficial effects of immunotherapies (e.g., IVIg, corticosteroids, mycophenolate mofetil, cyclophosphamide, and rituximab, alone or in combinations) have been reported recently [48]. In the same study, while half of the patients showed significant improvements or complete resolution of symptoms, the other half showed stabilization or mild improvement; although only a few showed progressive worsening of CAs.

Intrathecal injection of IgGs obtained from the patients induced ataxic gait in mice and the effects disappeared after the absorption of the Ab [43, 46], whereas administration into the flocculus impaired compensatory eye movements [51]. Notably, IgGs blocked the induction of PF-PC LTD in tissue slices [51], decreased mGluR1 clusters in cultured neurons [48], and blocked glutamate-stimulated formation of inositol phosphates in mGluR1α-expressing Chinese-hamster-ovary cells [46].

### Anti-GluR Delta Ab-Associated Cerebellar Ataxia: Clinical Profiles and Actions of Anti-GluR Delta Ab

GluR delta is a postsynaptic transmembrane protein localized at the PF-PC synapse [52]. Anti-GluR delta Ab has been described in non-paraneoplastic children [53–56] (Table 2). Usually, infection or vaccination precedes acute gait ataxia, associated with a variable degree of limb ataxia. Anti-GluR delta is positive in serum and CSF and CSF examination often shows pleocytosis, while MRI is usually unremarkable. Characteristically, these patients show good response to immunotherapy, such as IVIg and intravenous methylprednisolone (IVMP). Chronic recurrent CA, which shows a good response to corticosteroids, was also reported [57].

Actions of anti-GluR delta Ab were examined using monoclonal Ab against H2 ligand binding site of GluR delta, and not using Abs obtained from ataxic patients [58]. Subarachnoid injection of Ab against H2 ligand binding site of GluR delta elicited ataxic symptoms in mice and blocked the induction of LTD in cultured PCs [58].

### The Concept of LTDpathies

**Fig. 2:**
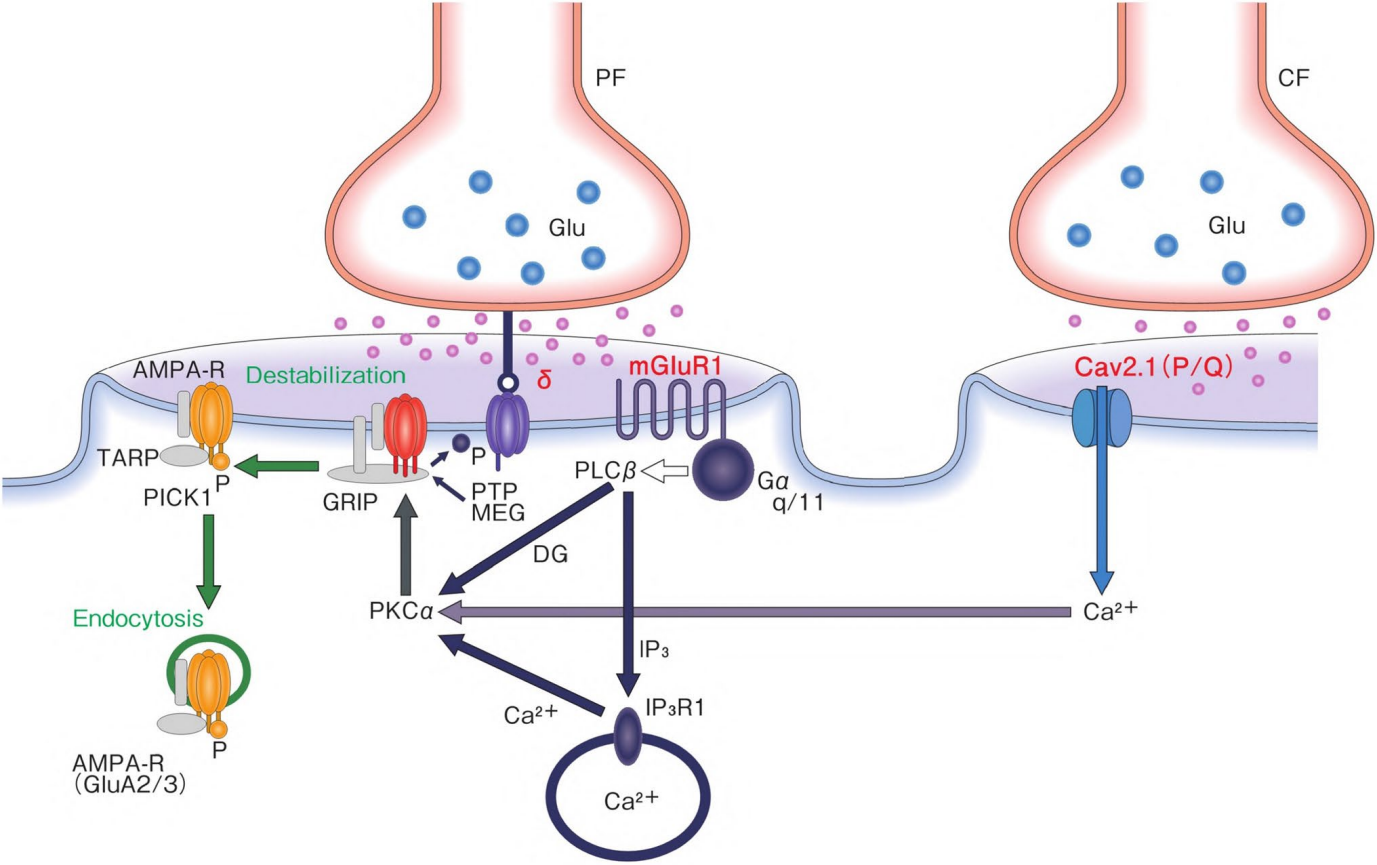
Schematic diagram of long-term depression (LTD) of excitatory synapses between parallel fibers and Purkinje cells. The climbing fiber input elicits complex spikes through the activation of dendritic P/Q type Ca^2+^ channels, leading to an increase in intracellular calcium concentration ([Ca^2+^]_i_). On the other hand, the parallel fiber input activates metabotropic glutamate receptor-PLCβ-IP_3_ signaling pathways, resulting in an increase in [Ca^2+^]_i._ The conjunctive activation of these two pathways increases [Ca^2+^]_i_ more than the additive level. The high [Ca^2+^]_i_ activates PKCα, and the latter phosphorylates GluA2 of the AMPA (α-Amino-3-hydroxy-5-methyl-4-isoxazolepropionic acid) receptor, which results in detachment of the AMPA receptor from scaffold proteins and its internalization with PICK1 in an AP2 and clathrin-dependent manner. CF, climbing fibers; PF, parallel fibers; Glu, glutamate; AMAPA-R, AMPA receptor; mGluR1, metabotropic glutamate receptor; Cav2.1 (P/Q), P/Q type Ca^2+^ voltage-gated channel; PLC, phospholipase C; PKC, protein kinase C; IP_3_, inositol triphosphate; GRIP, glutamate receptor interactive protein; TARP, transmembrane AMPA receptor regulatory proteins; PICK1, protein interacting with C kinase; δ, GluR delta 2; PTPMEG, megakaryocyte protein phosphatase

VGCC, mGluR 1, and GluR delta are involved in the induction of LTD between PF and PC (Fig. 2). Conjunctive activation of CF and PF induces LTD of PF-PC synapses on PCs [59–61]. CF activity increases [Ca^2+^]_i_ through the VGCC (P/Q-type) [62]. On the other hand, PF inputs in dendritic spines activate the mGluR-phospholipase C β(PLCβ)-inositol triphosphate (IP_3_) signaling pathway, which elicits Ca^2+^ release from the Ca^2+^-stores in the endoplasmic reticulum (ER) through IP_3_ receptors, and consequently increases [Ca^2+^]_i_ [63]. Simultaneous activation of these two pathways causes an increase in [Ca^2+^]_i_ level more than the additive level [64], which activates PKCα. The activated PKCα phosphorylates GluA2-C terminus, ultimately leading to the detachment of AMPA receptors from the scaffold protein and its internalization with PICK1 in AP2- and clathrin-dependent manners [65]. Under these situations, blockade of VGCC or mGluR1 will disturb the internalization of AMPA receptors. Consistently, anti-mGluR1 blocked the induction of PF-PC LTD and impaired adaptation of saccadic eye movements, a neural mechanism specific for the cerebellum [51].

On the other hand, the cytoplasmic terminus of GluR delta binds to megakaryocyte protein phosphatase (PTPMEG), which dephosphorylates tyrosine 876 in GluA2. Dephosphorylation of this site is necessary for the PKCα-induced phosphorylation of serine 880, an essential step in the internalization of AMPA receptors [66]. Thus, it is assumed that in addition to the roles of adhesion molecule, GluR delta serves to gate the induction of PF-PC LTD [66]. This assumption is supported by the finding that Abs against the N-terminus region (H2 ligand binding site) impair the induction of PF-PC LTD in cultured cells [58].

Taken together, Abs toward VGCC, mGluR 1, and GluR delta impair the induction of PF-PC LTD, which can disorganize PF-PC LTD-mediated cerebellar specific functions in CAs. In this regard, we have proposed the term “LTDpathies” to encompass all pathologies associated with dysregulation of PF-PC LTD [11, 67].

## Relevance of Synaptic Dysfunction with Impairments in Internal Forward Model

Anti-GAD65 and anti-VGCC Abs inevitably impair basal synaptic transmission [31, 44], while anti-mGluR1 and anti-GluR delta Abs interfere with excitatory synapses [58, 68]. The dysregulation in these deregulated basal synaptic transmissions is expected to weaken overall aspects of cerebellar functions. In addition, the auto-antibodies could also impair specific synaptic types of machinery, resulting in deficits in elementary cerebellar functions. The present review aims to discuss these pathophysiological mechanisms within a framework of physiological notions, online predictive controls, and motor learning.

The cerebellum serves as an online predictive controller [69, 70]. This section summarizes how auto-antibodies-induced synaptic dysfunctions impair the online predictive controls. To address these questions, we first review notions of predictive controls using an internal forward model, and summarize signal generation mechanisms in the cerebellum, “GABA-mediated disinhibition/inhibition mode,” for online predictive controls (the “GABA-Mediated Disinhibition/Inhibition Mode for Online Predictive Controls” section). Then, we show how auto-antibodies-induced dysfunctions are involved in this “GABA-mediated disinhibition/inhibition mode” section (the “Online Predictive Controls Disorganized by Auto-antibodies” section)”

Another specific cerebellar function is motor learning. The learning processes are facilitated by multiform synaptic plasticity [10, 11]. First, we discuss the relationship between learning processes and cerebellar reserve, capacities for compensation and restoration to pathologies (the “Synaptic Plasticity for Cerebellar Reserve” section), and then show how auto-antibodies impair the cerebellar synaptic plasticity, resulting in “a reduction in cerebellar reserve” (the “Impairment of Synaptic Plasticity Induced by Auto-antibodies” section).

### GABA-Mediated Disinhibition/Inhibition Mode for Online Predictive Controls

Temporal delays in sensory afferents reaching the CNS from the periphery set the signals from the feedback controller to result in oscillatory and unstable movements [69, 70]. To compensate for the delayed feedback controls, a predictive controller is embedded in the cerebellum for stable control of rapid movements [70]. This notion has been established based on various types of physiological experiments. In the task of fast goal-directed flexion movements that mimic the finger-to-nose test, ataxic patients show delay in the onset of antagonistic electromyographic (EMG) bursts associated with low rate of rise of EMG bursts, resulting in insufficient braking and overshoot from the target (*hypermetria, a cardinal symptom of cerebellar dysfunction*) [71–74]. The antagonistic bursts have the predictive nature of central origin [74]. In wrist-tracking tasks, movement of the wrist lags against the target in ataxic patients, resulting in irregular trajectories with intermittent corrections [75]. In these tracing movements, the predictive component in the movement showed increased error and delay compared to that of the controls [75].

The cerebellum performs the predictive calculation using an internal forward model [76], which solves the dynamics forward in time by combining the former state of the body from peripheral sensory organs, and the efference copy from the motor area in the brain [70, 77, 78]. In other words, the internal forward model serves as an internal feedback control. The cerebro-cerebellum receives projections from both the cerebral cortex and peripheral sensory pathways, which satisfy the basic requirements for the internal forward model [79]. Physiologically, PC simple spikes likely encode kinematic parameters, including velocity, position, and acceleration in random tracking tasks, allowing the cerebellum to correct the different parameters of movements [80–82]. The current output from the cerebellar circuit (dentate nucleus neurons: DNs) predicts future inputs to the cerebellum (mossy fibers) [83]. The above studies provide clear evidence for the cerebellum as a locus for the internal forward model.

**Fig. 3:**
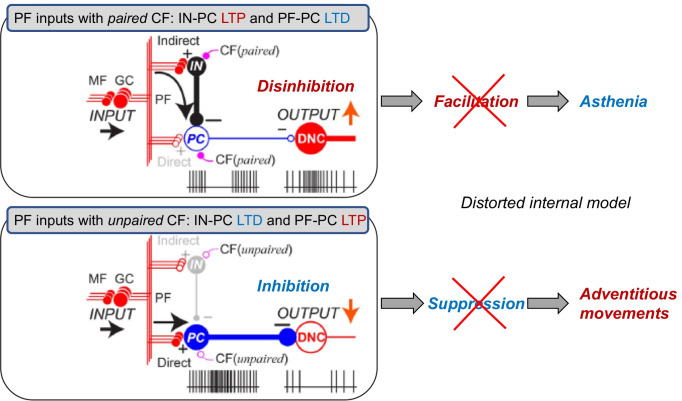
Possible cross-talk between synaptic plasticity and online predictive controls. Asthenia (top) is the consequence of the breakdown of the *Disinhibition mode* while adventitious movements (bottom) is the result of breakdown of the *Inhibition mode*. In the cerebellar cortex, mossy fiber (MF) inputs (INPUT) are relayed by granule cells (GCs) and processed through two parallel but different pathways: *Disinhibition mode* and *Inhibition mode*. Synaptic plasticity regulates the two modes. Parallel fiber (PF) inputs with climbing fiber (CF) activities induce LTP at interneurons (IN)-Purkinje cell (PC) synapses and LTD at PF-PC synapses, resulting in the *Disinhibition mode*. In contrast, PF inputs with unpaired CF induce LTD at IN-PC synapses and LTP at PF-PC synapses, resulting in *Inhibition mode*. In the *Disinhibition mode* (top), PF inputs activate INs that suppress PCs. Since PC activity provides tonic suppression of DNCs, its suppression facilitates DNCs through disinhibition (OUTPUT↑). A breakdown of this output mode leads to a decrease in facilitatory output, resulting in asthenia. In the *Inhibition mode* (bottom), PF inputs excite PCs directly. Because PCs are inhibitory, their activation suppresses the DNCs (OUTPUT↓). A breakdown of this output mode leads to a decrease in suppression, resulting in adventitious movements. (+): excitatory synapses, (−): inhibitory synapses. (Cited from ref. Ishikawa et al. 2015 [87])

Timing and synergy are target parameters in predictive computations of the internal forward model [70, 84–86]. The cerebellar output signals for timing and synergy controls are formed through a chain of inhibitory neurons, basket cells (inhibitory interneurons: IN), and PCs [34]. The majority of PCs, with somatosensory receptive fields (RFs) in the forearm, are suppressed before the onset of the wrist movements, and the majority of DNs with the same RF are concurrently activated. Thus, activation of DNs is generated by reduced inhibition by PCs, i.e., disinhibition (Fig. 3). In contrast, DNs with an RF in the proximal muscles not involved in wrist movements are suppressed by increased inhibition from PCs, i.e., inhibition (Fig. 3). Deficits in disinhibition cause a delay in the initiation of movements (timing delay), whereas deficits in inhibition elicit exaggerated activation in the muscles to be paused (asynergy). Notably, deficits of disinhibition and inhibition of DNs could be the physiological counterparts of *asthenia* and *adventitiousness*, respectively, the elementary symptoms described by Holmes (Fig. 3) [87, 88].

In conclusion, the cerebellum performs predictive computation of the internal forward model, where the output signals for predictive timing and synergy controls are formed in the fine-tuned temporal patterns using GABA-mediated disinhibition/inhibition mode.

### Online Predictive Controls Disorganized by Auto-antibodies

Anti-GAD65 Ab-induced reduction in GABA release causes dysfunction in a chain of GABAergic neurons, which leads to the disorganized disinhibition/inhibition mode and, thereby, inadequate operation of the online predictive controller. In this regard, the involvement of PF-PC LTD in the online predictive controls remains to be determined [89]. However, recent studies have shown that deficits in PF-PC LTD might elicit ataxic symptoms, presumably through the disorganized GABA-mediated disinhibition/inhibition mechanism. First, using the conditional knockout mice (a tetracycline-controlled gene expression system), acute blockade of mGluR impaired PF-PC LTD and simultaneously elicited motor incoordination without affecting basal synaptic transmission [68]. Second, a cross-talk between synaptic plasticity and online predictive control has been suggested [34]. CF activities reciprocally control the synaptic plasticity between PF-PC and IN-PC [90–94]. Stimulation of PF inputs paired with CF activities induced long-term potentiation (LTP) of IN-PC synapses and LTD of PF-PC synapses [34], whereas stimulation of PF inputs with unpaired CF activities induced LTD of IN-PC synapses and LTP of PF-PC synapses [34]. Thus, phasic suppression of PCs (i.e., disinhibition) is assumed to be facilitated by PF-PC LTD-induced attenuation of PF excitatory inputs (Fig. 3) [34].

In conclusion, the GABA-mediated disinhibition/inhibition mode is inadequately tuned in anti-GAD ataxia and presumably in LTDpathies, which might be one of the causes for the disorganized online predictive control in these diseases.

### Synaptic Plasticity for Cerebellar Reserve

Conjunctive stimulation of climbing fiber (CF) and PF induces LTD of PF-PC excitatory synapses [59–61]. It has been argued that error signals conveyed by CFs suppress persistently inadequate PF-mediated input efficacies and the long-lasting modification between PF synapses on PC constitutes the substrate for motor learning in vestibulo-ocular reflex, eyeblink conditioning, and adaptive adjustments of hand movement [95, 96]. On the other hand, since the first documentation of PF-PC LTD, multiple forms of synaptic plasticity in the cerebellar cortex have been documented. The cooperation of multiple forms of synaptic plasticity challenges the classic idea that single plasticity underlies a particular type of learning [97, 98]. Thus, others argue that the divergent forms of plasticity in the cerebellar cortex cooperate synergistically to ultimately create optimal output for behavior [97–99]. Accumulating evidence also argues against the view that CFs encode feedback error signals [100, 101].

**Fig. 4:**
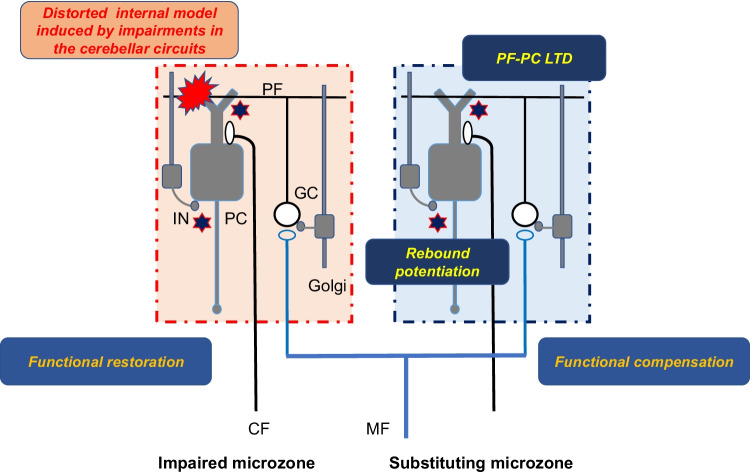
Functional restoration and compensation for the pathology. Anti-GAD65, VGCC, mGluR1, and GluR delta Abs functionally impair cerebellar circuits (for example, impairments in basal synaptic transmission), resulting in distortion of the internal model. For simplicity, the scheme assumes that impairments occur in one microzone (*left*). Functional restoration occurs in the impaired microzone, whereas functional compensation occurs in another substituting microzone. PF-PC LTD and rebound potentiation are involved in both mechanisms. MF, mossy fiber; CF, climbing fiber; GC, granule cell; PF, parallel fiber; PC, Purkinje cell; IN, inhibitory interneuron; LTD, long-term depression. White cells: excitatory neurons, gray cells: inhibitory neurons

It should be acknowledged that cerebellar synaptic plasticity has been considered solely in terms of learning. However, we have stressed another role for the synaptic plasticity in cerebellar reserve: the capacity for restoration and compensation to pathologies (Fig. 4) [102]. This capacity is the physiological background underlying clinical reversibility: a well-known clinical characteristic the ability of patients with transient cerebellar damage to show remarkable recovery [88]. Compensation and restoration result from rearrangement of remaining/undamaged synapses and subsequent reconstruction of the integrity of the cerebellar networks [103]. Thus, the multiple forms of synaptic plasticity play critical roles not only in the acquirement of the internal model in learning processes [104], but also update or repair the internal model after the pathology (Fig. 4). From the standpoint of therapeutic strategy, neuromodulation therapies, including noninvasive cerebellar stimulation and possibly transplantation in the near future, are recommended during the early stage when cerebellar reserve is preserved [105, 106].

In conclusion, the multiple forms of synaptic plasticity embedded in the cerebellum serve as a cellular mechanism of learning and a modulator between pathology and outcome. In the latter process, divergent types of synaptic plasticity appear to update or repair the internal model after the pathology.

### Impairment of Synaptic Plasticity Induced by Auto-antibodies

VGCC and mGluR1 are critical molecular components for increases in [Ca^2+^]_i_, which is an essential step in internalization of AMPA receptors in PF-PC LTD. In comparison, GluR delta plays modulator roles in the induction of PF-PC LTD. Auto-antibodies toward these molecules appear to dysregulate PF-PC LTD. In addition to the well-known PF-PC LTD, GABAergic synapses on PCs undergo long-lasting synaptic modifications. CF input elicits long-lasting potentiation of GABAergic synapses in PCs, termed rebound potentiation (RP) [107]. RP is mediated by VGCC (P/Q-type)-mediated increase in [Ca^2+^]_i_ [62]. The increase in [Ca^2+^]_i_ level activates Ca^2+^/calmodulin-dependent protein kinase II (CaMKII), which induces structural changes in GABA_A_R-associated protein (GABARAP) and subsequent enhancement of interaction between GABARAP and GABA_A_R γ2 subunit, leading to an increase in GABA_A_R expression at the inhibitory synaptic site [108]. Manipulation of inhibition of GABARAP and GABA_A_R γ2 subunit caused RP failure and lack of VOR adaptation [109]. Under these conditions, when GABA release is decreased, the postsynaptic enhancement effect in RP is diminished. Thus, anti-GAD Ab-induced suppression of GABA release can interfere with the induction of RP.

**Fig. 5:**
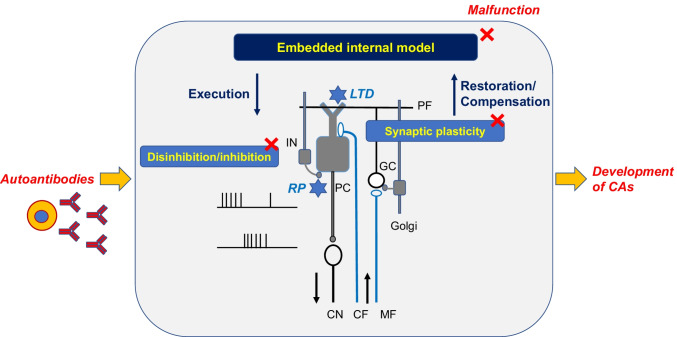
Pathomechanisms underlying cerebellar auto-antibody-induced synaptopathy. Accumulated experimental evidence suggest possible underlying mechanisms that link auto-antibodies-induced synaptic dysfunctions with manifestation of cerebellar ataxias (CAs). Auto-antibodies interfere with the machinery of GABA-mediated disinhibition/inhibition mode to distort the conversion process from the internal model to cerebellar output signals. Furthermore, these Abs also disorganize the induction of synaptic plasticity, long-term depression (LTD) and rebound potentiation (RP) on Purkinje cells (PCs), resulting in loss of restoration and compensation of the damaged internal model (loss of cerebellar reserve). These combinations of disorganized synaptic machineries will develop CAs. MF, mossy fiber; CF, climbing fiber; GC, granule cell; PF, parallel fiber; PC, Purkinje cell; Golgi, Golgi cell; IN, inhibitory interneuron; CN, cerebellar nucleus neuron. White cells: excitatory neurons, gray cells: inhibitory neurons

Taken together, anti-VGCC, anti-mGluR1, anti-GluR delta, and anti-GAD Abs prevent the induction of long-lasting synaptic plasticity on PCs. Each deficit in synaptic modification can disturb the reorganization of the internal model embedded in the cerebellar circuits: functional restoration and compensation for distorted internal model (Fig. 4). In other words, auto-antibodies-induced impairment of synaptic plasticity causes the loss of cerebellar reserve, which will amplify the functional damage by auto-antibodies and accelerate disease progression (Fig. 5). This mechanism might explain why CAs associated with these auto-antibodies are prominent compared with a degree of cerebellar atrophy.

## Structural Vulnerability of the Cerebellum and Neuroinflammation

In a forward model, motor and sensory inputs need to be integrated to elicit predictive outputs [70, 79]. Thus, abundant inputs from the cerebral cortex and the periphery converge on the PCs through mossy fibers (MFs) and PFs [110] and, consequently, PCs have numerous excitatory synapses (>>10^4^) [110]. These multimodalities are also the neural substrate for the cerebellar learning/reserve [102]. On the other hand, these structures can endanger the PCs through potential hyperexcitability, which could lead to cell death. Such vulnerability is amplified in the presence of cerebellar synaptopathy, in which GABA release is attenuated, or PF-PC LTD is dysregulated. Consistently, loss of PCs is evident in advanced stages of anti-GAD ataxia (see the “Synaptic Actions of Anti-GAD Ab” section).

**Fig. 6:**
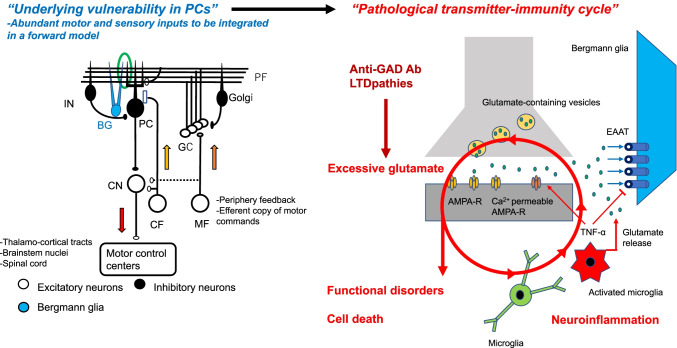
Underlying vulnerability in PCs and pathological transmitter-immunity cycle. Purkinje cells have an inherent vulnerability of excitotoxicity-induced cell death. Thus, auto-antibodies-induced synaptic dysfunction could elicit pathological transmitter-immunity cycle. In a forward model, abundant inputs such as periphery feedback and efferent copy of motor commands are necessary. This redundant information can be utilized for cerebellar learning and cerebellar reserve. Once glutamate level is excessive, microglia is activated, leading to the secretion of cytokines and glutamate release. TNF-α inhibits the uptake of glutamate on astrocytes and induces Ca^2+^ permeable AMPA receptors. Thus, the pathological transmitter-immunity cycle operates in an amplification manner, resulting in functional disorders or cell death. Anti-GAD Ab and, sometimes, Abs in LTDpathies could trigger the pathological transmitter-immunity cycle. The green circle in the left panel is magnified in the right panel. MF, mossy fiber; CF, climbing fiber; GC, granule cell; PF, parallel fiber; PC, Purkinje cell; Golgi, Golgi cell; IN, inhibitory interneuron; CN, cerebellar nucleus neuron, BG, Bergmann glia. White cells: excitatory neurons, Gray cells: inhibitory neurons, Blue: Bergmann glia. AMPA-R, AMPA receptor; EAAT, excitatory amino acid transporter

Under normal conditions, released glutamate is uptaken by astroglia, Bergmann glia in the cerebellum [111]. However, recent studies have shown that malfunction of microglia and astrocytes is involved in synaptopathy-induced cell death (Fig. 6). Excessive glutamate level activates microglia [112], which facilitates the release of glutamate from the microglia [113] and release of TNF-α [114]. TNF-α inhibits the re-uptake of glutamate through excitatory amino acid transporters (EAATs) on the astrocytes [115, 116], induces the expression of Ca^2+^ permeable AMPA receptors and NMDA receptors [115], and reduces the expression of GABA_A_ receptors on neurons [115]. Reactive astrocytes might be also involved the neuroinflammation and the BBB dysfunction, as observed in multiple sclerosis and experimental autoimmune encephalomyelitis [117, 118].

Therefore, cross-talk between glutamate and microglia/astrocytes might involve the positive feedback loop that accelerates excitotoxicity in cerebellar neurons. To clarify this mechanism of amplification, we propose the notion of pathological transmitter-immunity cycle (Fig. 6). In this cycle, excessive glutamate activates microglia, resulting in a release of cytokines for local neuroinflammation. These cytokines also impair the re-uptake of glutamate by astrocytes, leading to keep the cycle spinning. The transmitter-immune cycle that keeps rotating will induce functional disorders caused by an imbalance between glutamate and GABA, ultimately leading to excitotoxicity-induced cell death. The pathological transmitter-immunity cycle will be switched on by anti-GAD Ab and, sometimes, auto-antibodies in LTDpahties. Furthermore, this cycle might be involved in Glial fibrillary acidic protein (GFAP) astrocytopathy, an autoimmune astrocytopathy characterized by fever, headache, encephalopathy (ataxia, delirium, tremor, seizures, or psychiatric symptoms), and myelitis [119].

In conclusion, the convergence of inputs from the cerebral cortex and the periphery on the cerebellum is a neural substrate indispensable for cerebellar predictive control and cerebellar learning/reserve. However, these numerous inputs and excitatory synapses can increase PC vulnerability to excitotoxicity. The synaptopathy-induced excessive release of glutamate is amplified by the pathological transmitter-immunity cycle, which results in a pathophysiological switching from functional disorders to cell death.

## Conclusion

The studies reviewed here suggest that CAs associated with auto-antibodies against synaptic proteins can be explained by the dysfunction of a particular synaptic machinery with proper properties for delivering cerebellar specific functions. The synaptic dysfunction will lead to impairments in the internal forward model. Anti-GAD Ab interferes with the machinery of GABA-mediated disinhibition/inhibition mode to distort the conversion process from the internal model to cerebellar output signals. GABA-mediated disinhibition/inhibition might be also disorganized by deregulated LTD induced by anti-VGCC, anti-mGluR1, and anti-GluR delta Abs. Furthermore, these Abs also disorganize the induction of synaptic plasticity, RP, and LTD on PCs, resulting in loss of restoration and compensation of the damaged internal model (loss of cerebellar reserve), with subsequent worsening of CAs. The combination of these functional pathomechanisms might be operational in cerebellar synaptopathies.

This straightforward assumption should be validated in direct studies. First, we need to show that auto-antibodies from the patients interfere with synaptic plasticity, especially anti-GAD Ab for RP and anti-VGCC and anti-GluR delta Abs for PF-PC LTD. Second, the online predictive controls and tolerance to pathologies should be assessed under manipulation of GABA release and PF-PC LTD in in vivo preparations. Experiments should include assessments of the olivocerebellar pathways and the mossy fiber pathways, the 2 main inputs to the cerebellum. Third, there is a need to assess short-term, middle-term, and long-term consequences on the synaptic function, since reorganization of circuitry and proteins is likely. Fourth, there is a gap in our understanding of the prodromal phase which precedes the overt presentation of cerebellar symptoms. This should be extended not only to motor control studies, but also to cognitive, affective, and social domains. Fifth, the morphological and structural consequences of immune attacks on the cerebellar reserve deserve investigations, given the very high proportion of the neurons of the cerebellar circuitry and the option of improving cerebellar reserve to manage cerebellar disorders. Finally, molecules that are involved in the pathological transmitter-immunity circle should be determined for therapeutic strategies for avoiding cell death. Overall, there is a need for new techniques to be employed in these translational studies on cerebellar synaptopathies.

## Data Availability

The concept reported in this article is not based on raw data.

## Code Availability

There is no software application or custom code associated with the article.

## Abbreviations

Abs: antibodies
CaMKII: Ca^2+^/calmodulin-dependent protein kinase II
CAs: cerebellar ataxias
CF: climbing fiber
CSF: cerebrospinal fluid
DNs: dentate nucleus neurons
EAATs: excitatory amino acid transporters
EMG: electromyographic
GABARAP: GABA_A_R-associated protein
GAD65: glutamic acid decarboxylase 65
GAD67: glutamic acid decarboxylase 67
GluR delta: glutamic receptor delta
IMCAs: immune-mediated cerebellar ataxias ()
IN: inhibitory interneurons
IVIg: intravenous immunoglobulins
IP_3_: Inositol triphosphate
LESM: Lambert-Eaton myasthenia syndrome
LTD: long-term depression
LTP: long-term potentiation
MFs: mossy fibers
PCs: Purkinje cells
PFs: parallel fibers
PKC: protein kinase C
PLCβ: phospholipase C β
RFs: receptive fields
SCLS: small cell lung cancer
SPS: stiff-person syndrome
T1DM: type 1 diabetes mellitus
VGCC: voltage-gated Ca^2+^ channel
